# Preferred Attribute Elicitation (PAE) in the Sensory Descriptive Analysis of Foods: A Deep Comprehensive Review of the Method Steps, Application, Challenges, and Trends

**DOI:** 10.1111/1541-4337.70197

**Published:** 2025-05-15

**Authors:** Izabeli Batista Girarducci da Silva, Marciane Magnani, Erick Almeida Esmerino, Elson Rogerio Tavares Filho, Adriano Gomes Cruz, Tatiana Colombo Pimentel

**Affiliations:** ^1^ State University of Londrina (UEL) Londrina Brazil; ^2^ Laboratory of Microbial Process in Foods, Department of Food Engineering Federal University of Paraíba João Pessoa Brazil; ^3^ Fluminense Federal University (UFF) Niterói Rio de Janeiro Brazil; ^4^ Department of Food Federal Institute of Education, Science, and Technology of Rio de Janeiro Rio de Janeiro Brazil; ^5^ Federal Institute of Paraná (IFPR) Paranavaí Paraná Brazil

**Keywords:** attribute consensus, consumer evaluation, descriptive analysis, rapid sensory methods

## Abstract

Descriptive analysis (DA) is the gold standard for sensory profiling due to its robustness and reliability. However, its high cost, time demand, and labor intensity limit routine applications in the food industry. Rapid Sensory Profiling Techniques (RSPTs) have emerged as faster, less resource‐intensive alternatives to address these challenges. Among them, preferred attribute elicitation (PAE) stands out. This review aims, for the first time, to comprehensively clarify applications of PAE in food products and provide insights into challenges and trends. PAE enables consumers to collaboratively identify, measure, categorize, and prioritize key attributes while providing hedonic insight. By ranking attributes based on their relevance to product acceptance and preference, PAE integrates descriptive and affective dimensions of sensory perception, enhancing the understanding of consumer experience and product appeal. PAE has been applied to fruits, meat, dairy, dairy alternatives, bakery products, and beverages, yielding sensory profiles comparable to those obtained via DA. It has also been used to evaluate processing, resampling, storage, and fermentation effects, distinguish commercial products, and analyze sensory perception differences among consumer groups. Key methodological factors affecting PAE's reliability include the moderator's expertise, panelist profile, and attribute selection. Future research should expand its application to unexplored food categories, validate its relevance compared to traditional acceptance tests, assess its effectiveness in complex food matrices, and integrate it with other sensory methods. This is the first comprehensive review of the PAE application, offering practical insights for researchers and the food industry on correctly using this method.

## 1 Introduction

Sensory and Consumer Science, when applied to food products, is an interdisciplinary field that may involve, either jointly or separately, investigations in Sensory Analysis and Consumer‐Centric Studies (Jaeger et al. 2025). From the perspective of Sensory Analysis, the field measures how individuals perceive different attributes of appearance, aroma, flavor, and texture in food products through their senses of sight, smell, taste, touch, and hearing. Consumer Science examines factors such as attitudes, preferences, consumption habits, cultural influences, quality perceptions, label interpretation, and decision‐making processes (Civille et al. 2024; Stone et al. 2021).

Traditionally, sensory analysis methods are classified into three main categories. Discriminative methods aim to identify perceptible differences between products, employing techniques such as triangular tests, duo–trio tests, and paired comparisons. Descriptive methods seek to characterize the sensory properties of a product in detail, whether qualitatively, quantitatively, or both, to establish its sensory profile (J. Wang et al. 2024). Finally, affective methods assess the hedonic response of consumers, measuring their acceptance and preference for food products (Rodrigues et al. 2024).

Classical descriptive analysis (DA) methods, such as the Flavor Profile, quantitative descriptive analysis (QDA), Spectrum, and Texture Profile, are considered the gold standard for the description and profiling of food products (Stone et al. 2021; Civille et al. 2024). This is because such methods rely on highly specialized and trained panelists following rigorous protocols to ensure data repeatability and reproducibility. Their validation process guarantees that panelists can consistently reproduce their evaluations, accurately discriminate between different samples, and maintain a high level of agreement with other panel members. Applying these methods requires highly trained panelists, whose performance must be rigorously validated through statistical approaches aimed at ensuring robustness, repeatability, and reproducibility, guaranteeing the generated data's reliability (Tabary et al. 2021).

Despite their robustness and precision, the application of DA presents significant operational challenges. The extensive training required for panelists and the need for repeated analyses to ensure data reliability make these methods time‐consuming, labor‐intensive, and costly (Delarue and Lawlor 2022). These characteristics can hinder their routine implementation in the food industry, particularly in contexts that demand frequent analyses and rapid decision making in product development and quality control. Furthermore, although classical DA methods produce highly detailed and thorough product profiles, they may generate highly technical descriptions based on attributes not consciously perceived or processed by consumers during product selection and consumption (Ares and Jaeger 2013). In such cases, although analytically rigorous, the resulting sensory profiles may have limited relevance for consumer acceptance, which is often the ultimate goal of sensory evaluation in applied contexts today. This is because classical descriptive methods were not originally developed as tools to assess consumer acceptance. This association only gained popularity in the decades following their creation, particularly with the introduction of preference mapping (Greenhoff and MacFie 1994).

Due to the increasing demand for speed in the industry, primarily driven by a highly competitive environment, DA has undergone significant transformations in recent years. In this context, professionals and researchers in sensory analysis have been seeking more agile and accessible methods without compromising the usefulness of the generated information. In response to these limitations, Rapid Sensory Profiling Techniques (RSPTs) have emerged. They are designed to provide efficient, cost‐effective, and more accessible evaluations and are particularly well suited for consumer panels. Furthermore, they have gained popularity due to their practicality. Although they often yield less rigorous results due to the higher heterogeneity in consumer perceptions and lack of training, these methods are valued for their speed and efficiency.

RSPTs can be classified according to their theoretical core. First, there are methods based on verbalization (focused on the judgment of attributes that best describe each sample). In these methods, the samples are judged in a monadic and isolated manner; that is, no comparison is made between the samples for each attribute evaluated. Examples of these methods are the Flash Profile (FP), Check‐All‐That‐Apply (CATA), and preferred attribute elicitation (PAE). These techniques enable the rapid identification of relevant sensory attributes without requiring prior training. Second, there is a broader category of relative evaluation methods in which samples are assessed compared to each other. Within this category, two main subtypes can be identified. The first includes similarity‐based methods, such as Free Sorting, Projective Mapping (PM), and Napping, where panelists group or spatially arrange products based on their overall sensory perception. The second includes reference‐based methods, such as Polarized Sensory Positioning (PSP) and Pivot Profile (PP), in which products are evaluated relative to one or more predefined reference samples. Finally, dynamic methods, like Temporal Dominance of Sensations (TDS) and Temporal Check‐All‐That‐Apply (T‐CATA), assess how sensory perception evolves over time, making them particularly useful for products that undergo sensory changes during consumption (Delarue and Lawlor 2022).

Despite the advantages of rapid sensory evaluation methods—such as speed, lower cost, and ease of application—it is essential to acknowledge their limitations. First, the lack of formal training for participants can result in less precise descriptions and more significant variability in the data. The generated vocabulary tends to be spontaneous, often redundant, or poorly standardized, making interpreting results difficult and compromising comparability across studies. These methods generally show lower sensitivity in detecting subtle sensory differences between samples. Another critical issue are the nature of the data obtained, which are primarily nonquantitative, qualitative, or ordinal in nature, such as frequency counts (as in CATA) or relative rankings (as in FP), and the higher heterogeneity in consumer evaluations. In experiments involving many samples or samples with subtle or intense sensory characteristics (e.g., spicy, refreshing, or astringent products), rapid methods using consumers may not be ideal, as they tend to cause sensory fatigue and cognitive overload in participants. Sensory fatigue and cognitive overload may also occur with trained panels, but training may improve the ability of assessors to identify subtle differences. Furthermore, there are challenges regarding the reproducibility and reliability of results, as these methods lack the familiarization and training steps used in classical approaches and often reflect the local context of the consumer population (Valentin et al. 2012).

In this context, PAE has successfully obtained sensory profiles, standing out for its consumer‐centered approach. The PAE method has been proposed as a rapid technique with the advantage of a hybrid approach, it is not only focused on product description but also on the consumer's affective response to that product. PAE allows consumers to elicit and rate the intensity of sensory attributes that influence their acceptance and perception of a product. Thus, the method integrates both descriptive and hedonic components, offering an integrated view of the consumer's sensory experience. Its ability to generate relevant data from the consumer's perspective, without the need for trained panelists, makes PAE particularly attractive to the food industry and agile product development processes (Grygorczyk et al. 2013; da Costa et al. 2020; J. M. da Silva et al. 2021).

The PAE method has been increasingly applied in sensory studies over the past decade (Bezerril et al. 2022; Canella et al. 2024; da Costa et al. 2020; Fernandes et al. 2021; J. M. da Silva et al. 2021). Bezerril et al. (2022) observed using PAE that the addition of xique‐xique jam to yogurt—either alone or combined with prebiotics—resulted in higher intensities of fermented aroma, herbal aroma, and herbal flavor, whereas the control sample sweetened with sucrose showed higher intensities of goat and cheesy aromas. Canella et al. (2024) evaluated fermented milks and observed, using PAE, that samples formulated with skimmed milk, 6% inulin, and probiotics, as well as those made with whole milk and probiotics, showed higher sensory acceptance compared to the sample produced with skimmed milk concentrated by freezing. The sample with concentrated skimmed milk was more associated with intense goat flavor, acidity, yellowish color, higher viscosity, and saltiness—attributes that contributed to its lower acceptance. da Costa et al. (2020) evaluated probiotic yogurts using PAE and reported that the three main attributes driving liking were sweet taste, creaminess, and consistency. Fernandes et al. (2021), in turn, reported that the typical flavor of fermented almond beverages was more important than the almond flavor itself, and that liking was negatively affected by samples with a milky or watery appearance and sandy texture. Interestingly, the authors noted that there was no consensus on the ideal consistency, indicating different expectations: some consumers preferred a thicker beverage, similar to yogurt, while others expected a more liquid product. Finally, J. M. da Silva (2021) evaluated frozen desserts made with water‐soluble extract from rice byproducts and enriched with prebiotics, and reported that nine out of the 13 attributes elicited by participants—such as yellow color, brightness, creamy appearance, passion fruit aroma and flavor, sweet, acidic, and sour taste, and creamy texture—were considered important by both vegan and nonvegan consumers, although the order of importance varied between groups. For vegans, the most relevant attributes were the passion fruit flavor and aroma, as well as the consistency and texture of the product, reflecting greater attention to naturalness and structural characteristics. Nonvegans, on the other hand, prioritized yellow color, sweet taste, and creamy appearance, with greater emphasis on visual and flavor aspects. However, no comprehensive review has been conducted to examine PAE application, rationale, and methodological relevance in sensory DA of food products. Therefore, this review aims to bridge this gap by providing a detailed overview of PAE as a sensory descriptive tool for food products, discussing its applications, challenges, and emerging trends.

## 2 DA: From Classical to Rapid Methods

### 2.1 Classical DA

DA is the gold standard approach for obtaining the sensory profile of food products. It provides a detailed description of all attributes present in food products, whether qualitative, quantitative, or both, enabling their comparison based on different attributes (McSweeney 2024).

The origins of DA stem from the need for a structured method to objectively and reproducibly describe sensory characteristics. Historically, DA emerged to categorize sensory methods designed to describe samples, with four main techniques: FP, Texture Profile Method (TPM), QDA, and Spectrum. The FP was introduced in the 1950s and involved panelists led by a trained coordinator, who described products using an agreed‐upon terminology and a nonnumerical scale, relying on consensus (Cairncross and Sjostrom 2004).

Another major advancement in DA was the development of the TPM, introduced by Brandt, Skinner, and Coleman (Brandt et al. 1963) in the early 1960s. Based on the principles of the FP method, TPM represented the first comprehensive attempt to systematize the sensory evaluation of texture. The method defined texture in terms of mechanical, geometrical, fat‐related, and moisture‐related characteristics, evaluated in the sequence in which they are perceived—from the first bite, through mastication, to residual sensations. TPM established a structured methodological framework using standardized scales and specific training protocols, enabling qualitative and quantitative texture characterization.

Later, in the early 1970s, QDA was introduced, modifying FP by implementing linear scales for quantification and eliminating consensus‐based analysis, except in the initial attribute elicitation phase. With a focus on panelist calibration to achieve high repeatability and reproducibility, QDA supports using parametric statistical analyses in sensory studies (Sidel et al. 2017). QDA also introduced methodological innovations, such as using reference materials during training sessions to calibrate assessors until individual and group repeatability is achieved for each attribute. Only after demonstrating good performance and repeatability do the panelists proceed to the actual evaluations conducted without references. The judgments are made independently during the evaluation phase, emphasizing individual perception rather than group consensus. Another distinctive feature of QDA is its focus on relative differences among products rather than absolute quantification of attributes. As is widely recognized, humans have difficulty judging absolute values but are pretty effective at perceiving and comparing relative differences (Stone et al. 2021).

The Spectrum method, introduced in the late 1970s, differentiated itself using an absolute scale ranging from 0 to 15, measured in tenths, providing 151 discrimination points. In addition, Spectrum introduced attribute‐specific training references beyond the anchor‐based training proposed by QDA and encouraged using standardized lexicons rather than generating terms by consensus (Dus et al. 2017).

Despite methodological differences, these classical descriptive techniques share a common structural framework. The process typically begins with experimental design, which involves defining the number of potential participants (usually between 25 and 30) to select the final panel (normally 8–12 panelists), determining the number of replications (at least three to ensure statistical reliability), and controlling carryover effects by implementing appropriate palate cleansers. The number of samples per session is also carefully considered to minimize sensory fatigue among panelists. These techniques also share methodological characteristics that significantly contribute to their robustness, such as the exhaustive pursuit of consensus on the definition of each attribute and its intensity range, as well as the repeatability of assessors, which allows for the generation of highly uniform sensory descriptions. This results from rigorous training with standardized scales, during which assessors taste products and reference samples for each attribute until they form a memory of the corresponding intensity range. This process leads to greater sensitivity in detecting differences between samples, making it easier to assess subtle intensity variations. In addition, using triplicates or even quadruplicates in evaluations helps reduce random error (Civille et al. 2024).

In the panelist selection stage, motivation and commitment are assessed, followed by sensory acuity screening using basic taste and odor recognition, threshold perception, ranking tests, and triangular tests with sequential analysis (Ray 2021). Once panelists with the required sensory acuity are identified, they proceed to the attribute generation phase, where descriptor terms specific to the product under evaluation are determined, along with reference standards representing their intensity. This phase is crucial and can be conducted through consensus, voting, or structured methodologies such as Repertory Grid (Castanho et al. 2024). Reference standards ensure consistency in evaluations and include physical/chemical references (chemical compounds, real food products, and ingredients) for verbal attribute anchors.

The training and validation phase begins after identifying panelists and reference standards. During this stage, panelists undergo multiple sessions to familiarize themselves with the data collection instrument, the reference anchors for each attribute, and the product samples to be evaluated. Most descriptive panel training programs require between 40 and 120 h of training, depending on panelist experience, product complexity, and the degree of difference between samples (Civille et al. 2024). To avoid overestimating the time requirements for sensory training, it is important to distinguish between panel development hours, which refer to the initial training of individuals to become qualified descriptive assessors, and project‐specific training, which involves calibrating already trained panelists for a particular product category. Blind tests are often conducted to ensure panelists correctly recognize reference standards without external influence. Statistical analysis of training data is performed to verify panel consistency and discriminatory ability.

For instance, QDA offers a significant advantage through its robust validation process. After each test, individual panelist performance is assessed using one‐way analysis of variance (ANOVA) for each attribute, ensuring that panelists can reliably differentiate products. This step minimizes variability in human sensory perception and reduces dependence on a small group of potentially atypical responders. In addition, QDA accounts for product variability, recognizing its potential influence on panelist responses. Employing repeated measures designs and two‐way ANOVA effectively partitions subject and product effects, improving the understanding of product differences and individual discriminatory capabilities (Stone et al. 2021).

Once panel training and validation are complete, ensuring uniformity in perception and evaluation accuracy, DA proceeds to its crucial data collection phase. During this stage, trained panelists assess product samples using unstructured 9‐ or 15‐cm scales to rate the predefined sensory attributes, relying on reference standards from training. Each panelist assigns intensity ratings for each attribute in each sample (Stone et al. 2021). Ensuring controlled testing conditions is essential to minimize external influences that could be potential biases.

The collected data undergo rigorous statistical analysis, typically involving parametric and nonparametric methods such as ANOVA, principal component analysis (PCA), and Cluster Analysis to identify key sensory attributes and significant differences between samples (J. Wang et al. 2024).

Classical DA methods present important strengths that support their establishment as the standard for profiling in sensory science. Among their advantages is the ability to generate comprehensive descriptions, trace attributes associated with sensory defects and analyze many attributes without causing fatigue to the panelist. They also allow for modality‐specific analyses, providing quantitative and qualitative data with high precision, reproducibility, and traceability. Using trained panelists enhances sensitivity in detecting subtle sensory differences, while structured protocols and replication contribute to the reliability and repeatability of results. Furthermore, DA methods facilitate the development of standardized sensory vocabularies linked to ingredients or process variables, providing valuable insights for product development, quality control, and benchmarking. Their strong internal validity, especially with robust parametric statistical analyses such as ANOVA and multivariate techniques, makes these methods particularly suitable for hypothesis‐driven research and sample optimization. This way, the main advantages of DA include its validity and robustness in describing and quantifying sensory attributes, enabling precise differentiation of complex or similar products (Koushki et al. 2024).

However, this methodology presents challenges, such as high costs, long training periods, and the complexity of implementing a strict protocol, making it less accessible for the food industry, where rapid product development is often required (Ribeiro et al. 2023).

Rapid sensory methods have been developed to address these limitations, utilizing consumer panels or partially trained assessors to generate quicker, more cost‐effective, and more flexible results (Rodrigues et al. 2024).

### 2.2 Rapid Methods for Descriptive Profiling

In response to industry demands for more efficient and cost‐effective DA techniques, various alternative methods have been developed to complement traditional profiling approaches, such as DA (Valentin et al. 2012). The RSPT can be categorized according to the theoretical foundation of the method, such as verbal description, relative evaluation methods, and dynamic temporal evolution (Delarue and Lawlor 2022), as detailed in the introduction of this article (Section 1). However, they can also be classified according to their methodological core: the type of task required from the consumer and the nature of the data extracted (structured vs. unstructured) (Ribeiro et al. 2024).

Methods that rely on predefined descriptive terms include Rate All That Apply (RATA), CATA, and Just About Right (JAR). These methods use a predefined list of sensory attributes derived from previous literature on widely studied products or established through elicitation techniques such as Repertory Grid (Galler et al. 2020; Lee et al. 2023). In the CATA method, consumers do not indicate the intensity of their perceptions but instead, select the attributes from the list they recognize in the evaluated sample. The RATA method follows the same logic; however, consumers must also indicate the perceived intensity for all attributes identified in the sample. In the JAR method, consumers assess the extent to which the intensity of the listed attributes approaches the ideal intensity level for the given product (Ribeiro et al. 2024).

Represented by PSP and PP, the methods based on comparisons with references, use reference samples to position other samples sensory‐wise. In PSP, a set of reference samples serves as anchoring poles, and assessors position the samples based on perceived similarity to these poles (Teillet et al. 2022). In PP, a pivot sample is used as a reference point, with all other samples being evaluated concerning this central reference (S. Wang et al. 2023).

The methods based on global differences and similarities include PM/Napping and Sorting. PM/Napping relies on distributing samples in a bidimensional space, considering their similarities and differences intuitively. In Sorting, participants group samples according to their sensory perceptions. This can be done as an open process, where assessors define the groups freely, or as a guided process with predefined categories.

Finally, methods based on free description include FP, Free Listing (FL), Free Comment (FC), and PAE. In FP, assessors individually create a preliminary list of attributes by comparing samples, focusing on perceived differences without using hedonic terms. They then receive a consolidated list of all generated attributes and adjust their own lists accordingly. Finally, they rank the samples for each attribute based on perceived intensity, allowing for ties when necessary (Delarue and Lawlor 2022).

The FL method involves the spontaneous listing of perceived attributes in a product, followed by successive tasks of classifying and comparing samples based on these attributes. In contrast, FC focuses on obtaining free comments without requiring sample comparison or positioning. It is a cognitively less demanding and more intuitive method, often not restricted to the sensory description of the evaluated samples. To mitigate the generation of many low‐interpretation‐value terms, researchers usually instruct assessors to focus on sensory modalities, for example, by directing the analysis with phrases such as “Please describe the visual aspect of this ham” (Mahieu et al. 2022).

More specifically, as the focal method of this review, in PAE, consumers assess product acceptance using a nine‐point hedonic scale, considering appearance, aroma, taste, and texture. After this evaluation, participants record the attributes associated with their perceived product acceptance or rejection. These attributes are then discussed in a group with a research moderator, who organizes them into a visual board, allowing for the identification of common terms and the elimination of redundancies by grouping synonyms and opposite terms into the same scale (e.g., acidic and sour; salty and bland).

After attribute elicitation, the anchor terms for each attribute are defined, generally ranging from None/Extremely Weak to Extremely Strong. Next, panelists rank the attributes based on their importance for product acceptance, prioritizing the most relevant sensory factors. Finally, after a short break, participants reassess the samples and record attribute intensity based on previously defined anchors (J. M. da Silva et al. 2021).

For most rapid methods, sensory maps that closely resemble those obtained through PCA in DA can be generated using different statistical approaches. However, the statistical evaluation of data from these methods may be challenging. Various approaches are used depending on the method, such as the Cochran Q test, GPA, multiple factor analysis (MFA), correspondence analysis (CA), multidimensional scaling (MDS), and hierarchical clustering analysis (HCA), among others (Marques et al., 2022).

## 3 Review Methodology: Scoping Review on PAE Application to Food Products

In this review, we adopted the Scoping Review framework proposed by Arksey and O'Malley (2005), with adaptations suggested by Levac et al. (2010), including refinement of the research question with a clearly defined scope, adoption of an iterative and team‐based process for study selection and data extraction, combined numeric and thematic synthesis, and an emphasis on the interpretation of findings and consultation with stakeholders as a step toward knowledge translation. The review protocol was structured into five phases.

In the first phase, we formalized the research question, aiming to provide a comprehensive overview of how the PAE method has been applied to food products. In other words, there was no directional hypothesis or specific problem statement, but rather a broad question: “How has PAE been used in food products?” The broad nature of the question was guided by the fact that the application of this relatively new method is still emerging, and it has not yet been widely applied, which allowed for an exhaustive analysis of its use to date.

The second phase involved selecting databases and defining the search strategy. The literature search was conducted in the ScienceDirect, Web of Science, and Scopus databases, including all studies published up to January 26, 2025. To ensure comprehensive retrieval of relevant studies in the food sector, keywords were combined using a structured search operation: ALL (“preferred attribute elicitation”) in Scopus, which yielded 179 records; “preferred attribute elicitation” in the general search field of ScienceDirect, which returned 32 results; and “preferred attribute elicitation” (All fields) in Web of Science, which returned 11 records.

In the third phase (study selection), all retrieved records were imported into Mendeley, the reference management software used in this study. Duplicate records were automatically identified and removed. The remaining studies underwent a three‐step screening process. In the first step, two independent reviewers (authors of this study) screened titles and abstracts, excluding those not aligned with the scope of the review. In the second step, full‐text readings were conducted to verify adherence to the preestablished inclusion and exclusion criteria. Any reviewer disagreements were resolved with support from a third researcher, ensuring consensus on final inclusion or exclusion decisions. A citation tracking technique was also used to identify and include relevant studies cited in the initially selected articles but not retrieved in the database searches. Studies from related fields that emerged during the screening process were considered theoretical support only and not included in the final synthesis.

Inclusion criteria required that studies explicitly address the use of PAE in food products. Studies without a clear connection to PAE, conference abstracts, editorials, and studies lacking a detailed methodology were excluded.

In the fourth phase, relevant information from each study was extracted based on the PICOS strategy (Methley et al. 2014), adapted to the food science field in which this review is situated. Data were collected regarding the population involved in the sensory tests; the intervention was defined as the steps used in the PAE method and its intended purpose; the comparison elements were the samples evaluated in each study; and outcomes were defined as the results observed by the authors, as well as the methodological design adopted. This standardized extraction allowed for a more structured comparative analysis across studies, facilitating the identification of patterns, gaps, and relevant contributions to the objectives of this review. In the fifth phase, the results were synthesized using a framework‐based thematic analysis model, considering three main dimensions: (1) the methodological steps of PAE in each study, (2) the type of product analyzed, and (3) the intended purpose of using PAE.

## 4 Results

### 4.1 Method Application and Statistical Analysis

PAE is a rapid descriptive method initially introduced at the 5th Pangborn Sensory Science Symposium in Boston, MA 2003. The presentation was titled “Understanding consumer perceptions, attitudes, and choices for soymilk beverages.” However, the first article was only published in 2013 (Grygorczyk et al. 2013). To summarize the proposed method, consumers first individually assess their liking or disliking of a product's sensory attributes using a nine‐point hedonic scale and describe the perceived attributes, with the option to indicate those influencing their judgment. The method then shifts to a collective phase, where a research moderator guides the nomination and grouping of attributes to reduce redundancy and collaboratively establishes scale anchors. Consumers then evaluate attribute intensity using a verbally anchored seven‐ or nine‐point structured scale and rank their importance in product acceptance. Finally, after a brief pause, they reassess the attributes monadically in the samples.

**FIGURE 1:**
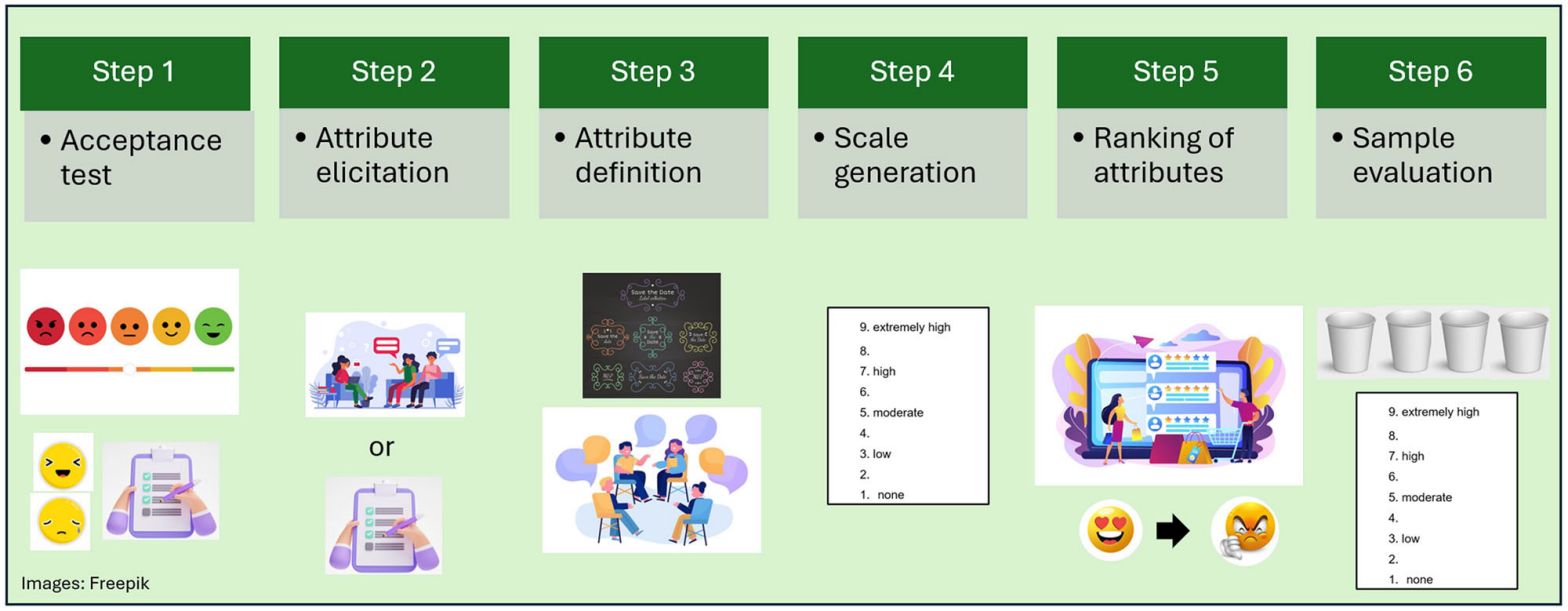
Preferred attribute elicitation (PAE) steps for application in food products.

Figure 1 presents the PAE steps for application in food products considering the published articles. In a general view, the method is applied in six steps. In the first step (acceptance test), the participants receive the samples in a monadic form and are requested to denote their acceptance using hedonic scales (usually a nine‐point hedonic scale). In this step, they also may be asked to write down what attributes they liked or disliked, aiming to encourage the participants to think about the product's attributes (Auriema et al. 2021; Grygorczyk et al. 2013; McSweeney et al. 2016, 2017; Muggah and McSweeney 2017). The purchase intention may also be evaluated (da Silveira Maia et al. 2025). The acceptance test is performed before the DA of the samples to avoid the halo effect (Grygorczyk et al. 2013).

In the second step (attribute elicitation), the participants receive all the samples simultaneously. They are asked to name/verbalize or list/write down all the attributes they consider important for the product's characterization or acceptance (de Assis et al. 2024; Bezerril et al. 2022; da Costa et al. 2020; da Silveira Maia et al. 2025; J. M. da Silva et al. 2021) or that differentiate the samples (Auriema et al. 2021; Grygorczyk et al. 2013). Most of the studies (de Assis et al. 2024; Bezerril et al. 2022; da Costa et al. 2020; Dias et al. 2023; Fernandes et al. 2021; Luciano et al. 2023; da Silveira Maia et al. 2025; de Oliveira et al. 2024; Popoola et al. 2019; Rocha et al. 2023; J. M. da Silva et al. 2021) asked the consumers to list/write down the attributes individually, which may result in attributes from all participants. This step is important to motivate participants to be thoughtful about the key attributes that stood out and contributed to the perception or acceptance of a product. By developing their own opinions, participants can effectively participate in group discussions during the following stages, ultimately aiding in the characterization of the product by the PAE. If the elicitation is performed verbally, the moderator participates in this step (Muggah and McSweeney 2017).

A moderator guides the consumer group on steps 3 and 4. The moderator selection is important, as this person will encourage discussion on the generated attributes and scale anchor terms, foster engagement and attribute consensus, ensure that the method is correctly performed, and the attribute evaluation form is addressed. Therefore, the moderator should facilitate an in‐depth discussion on the attributes and anchor scales and guide the group. The dominance of some participants during these steps may occur, and the moderator may need to guide the activities to obtain the opinions of all members of the groups (Ribeiro et al. 2025). Finally, the moderator should create an environment that encourages consumers to state their perceived attributes honestly and with respect and collaboration (Ribeiro et al. 2025).

In step 3 (attribute definition), the attributes elicited by the participants are written on a board and grouped according to the manner the participants consider appropriate. Usually, the attributes are grouped in appearance, aroma, flavor, and texture (de Assis et al. 2024; Luciano et al. 2023; da Silveira Maia et al. 2025; J. M. da Silva et al. 2021). Then, a discussion is performed aiming to group attributes with similar meanings and excludes those that are not consensus among the group or challenging to evaluate. For that, the moderator notes the attributes that could have identical meanings and discusses with the group if they effectively assess the same product characteristics and which term the consumers consider more appropriate to use. Furthermore, all attributes are mentioned, and their importance for product characterization is discussed to identify those without consensus and exclude those the consumers consider inadequate. At this stage, attributes are also narrowed down further by asking questions such as, ‘‘Do any more of these attributes mean the same thing to everyone?’’ and ’’Are there any attributes here that you feel you won't be able to easily evaluate in the product?’’ (Grygorczyk et al. 2013).

In step 4 (scale generation), the participants define the terms describing the ends of the intensity scales for each attribute. Generally, 7‐ (Grygorczyk et al. 2013; McSweeney et al. 2016, 2017; Muggah and McSweeney 2017), 9‐ (de Assis et al. 2024; Auriema et al. 2021; Bezerril et al. 2022; da Costa et al. 2020; Fernandes et al. 2021; Luciano et al. 2023; da Silveira Maia et al. 2025; J. M. da Silva et al. 2021), or 10‐point scales (Canella et al. 2024) (1 = few/low, and 7 or 9 = a lot/high) are used. In this step, the participants can still include or exclude attributes if necessary. Furthermore, the evaluation form for each attribute is defined. To date, we could observe a higher prominence of studies using nine‐point intensity scales, which may be related to the participant's familiarization with nine‐point hedonic scales, facilitating their utilization with untrained panelists. The choice of scales may impact on the results, but up to date, no previous study compared different scale lengths in PAE application. In this way, other scale sizes could be evaluated, such as shorter scales, as usually used in other methods, such as RATA.

In step 5 (ranking of attributes), the participants are asked to rank the attributes according to their importance for acceptance of the samples (from the most important to the least important). Participants are advised that equally important attributes could have the same ranking order. The ranking task was performed in groups (verbally) or individually (using paper ballots). Most of the studies (de Assis et al. 2024; Bezerril et al. 2022; da Costa et al. 2020; Dias et al. 2023; Fernandes et al. 2021; Luciano et al. 2023; da Silveira Maia et al. 2025; de Oliveira et al. 2024; Popoola et al. 2019; Rocha et al. 2023; J. M. da Silva et al. 2021) asked the consumers to rank the attributes individually, which may generate results that can be statistically analyzed and come from the perception of each of the participants. Some participants could be prominent when the group elicits the ranking of the attributes. Finally, after a break (usually 5–35 min), in step 6 (samples evaluation), the participants receive the evaluation sheet and samples in a monadic form, and they are asked to evaluate the intensity of each attribute using the scales they had chosen. The methodology was presented using paper ballots for attribute elicitation and an evaluation sheet for attribute intensity evaluation because this form was used in all studies. However, it is important to mention that these steps could be performed using computers.

Some variations of the method have been performed. Some studies do not include the acceptance step because the objective is only evaluating the differences in the sensory attribute intensity (de Assis et al. 2024; Auriema et al. 2021; Dias et al. 2023; Luciano et al. 2023) or because acceptance tests with a higher number of consumers are performed separately (J. M. da Silva et al. 2021; T. O. Silva et al. 2024; Soares et al. 2020). Furthermore, some studies did not include the ranking of attributes step (Auriema et al. 2021; Bezerril et al. 2022; Canella et al. 2024; Dias et al. 2023; Luciano et al. 2023). In addition, most of the studies evaluated all the characteristics of the samples (appearance, aroma, flavor, and texture) (de Assis et al. 2024; Auriema et al. 2021; da Costa et al. 2020; Dias et al. 2023; Fernandes et al. 2021; Luciano et al. 2023) but some studies limited the attributes type such as only aroma and flavor (Bezerril et al. 2022), texture (Grygorczyk et al. 2013), flavor (McSweeney et al. 2017), and aroma, flavor, and appearance (de Oliveira et al. 2024).

Therefore, we provide a general overview of PAE application based on previous studies. However, changes may be performed considering the objective of each study and its experimental design. It is important to note, however, that most changes in previous studies were based on the exclusion of some steps unrelated to the descriptive task (acceptance test or ranking test), which, in a general view, will not impact the application of the method and the results. However, other authors preferred to include all steps and not use the acceptance data, for example, aiming not to modify the original method (Muggah and McSweeney 2017). It is important to highlight that the removal of the acceptance test and ranking test turns the methodology into a DA using consumers (untrained assessors), which would not provide insight into impactful attributes, which is the main advantage of this methodology. In contrast, the narrowing of an attribute type (only aroma, flavor, or texture) should be interpreted cautiously, as a previous study reported a lack of correlation between PAE and DA results when considering only the flavor of cookies (McSweeney et al. 2017). If other changes are performed (changes in attribute discussion and consensus or scale type, for example), the impact on the method performance must be evaluated.

The PAE data for the intensity of attributes are analyzed using GPA. GPA analysis can reduce the scale use effect, generate a consensus configuration, and permit comparisons of the attributes proximity that different consumers use to characterize the samples (Soares et al. 2020). It is considered a multivariate exploratory methodology that uses transformations (translation, rotation, isotropic rescaling, and reflection) of the individual assessments to provide optimal comparability (Muggah and McSweeney 2017). Furthermore, it is possible to compare the different panelists with each other and plot the individual panelists' data spaces (Lawless and Heymann 2010).

**FIGURE 2:**
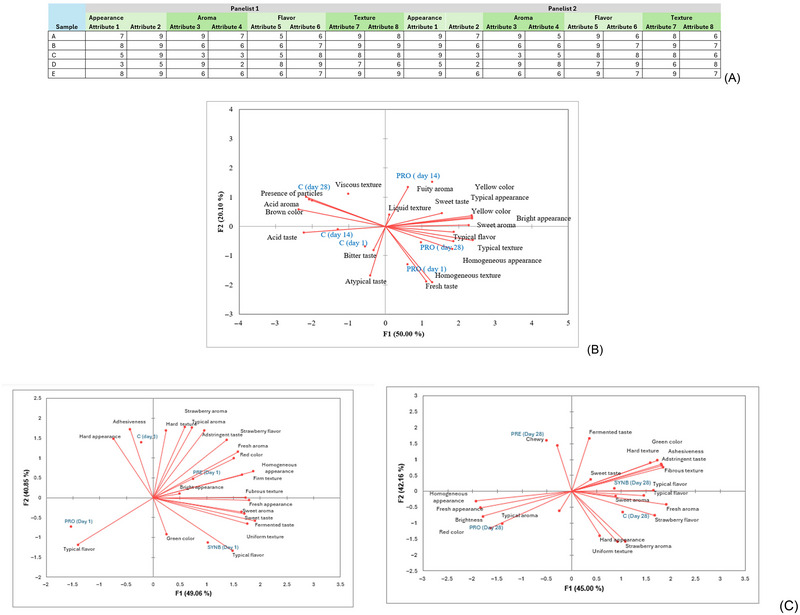
Statistical approaches for preferred attribute elicitation (PAE) data. (A) Data matrix, (B) GPA for evaluation of storage time in one section, and (C) GPA for evaluation of storage time in sections at the storage intervals. Data prepared for this review for didactic purposes.

For GPA analysis, a data matrix is constructed considering the samples in rows and the intensity of the attributes for each participant in the column (Figure 2A). Therefore, the number of columns will be the number of consumers multiplied by the number of attributes. Usually, the number of factors considered in the GPA maps is defined by using factors with eigenvalues higher than 1, a minimum prespecified variance explanation (70%, 80%, or 85%), or that makes sense based on the knowledge of the subject. However, no more than three factors are usually maintained due to difficulties in interpretation (Lawless and Heymann 2010). After that, the attributes with a correlation with the axis higher than 0.6–0.7 (positively or negatively) are considered significant in describing the sensory characteristics of the samples and are used for discussion (R.Silva et al. 2020). A previous study also used HCA as a complementary approach to GPA, as the dendrogram obtained in HCA may facilitate the visualization of differences in the sensory characteristics of the samples based on group formation (R. Silva et al. 2020).

However, it is important to note that a previous study used PCA to evaluate PAE data (Viana et al. 2024), which is not recommended because untrained panelists and structured scales are used in PAE. PCA assumes that each participant employs the sensory attributes in the same form and describes differences between samples using the same range values (Hunter and Muir 1995), which may occur only using trained participants and unstructured scales. Therefore, PCA may be used for DA but not for PAE. GPA equalizes attributes and scale usage, and instead of using mean values like PCA, it uses individual scores to account for variance. Since all panelists evaluate the same samples, the samples remain constant and do not vary. We highlight here that using nonrecommended statistical analysis may result in biased results. In addition, the publication of results with wrong statistical analysis may induce readers to repeat the wrong protocols.

The ranking of the important attributes for sensory acceptance has been analyzed using the Friedman test (de Assis et al. 2024; da Costa et al. 2020; Fernandes et al. 2021; de Oliveira et al. 2024). However, it is essential to note that many studies do not mention the statistical approach used for this step or do not present the results (Grygorczyk et al. 2013; McSweeney et al. 2016, 2017; Muggah and McSweeney 2017). This is related to the differences in the application of the ranking task. In cases where all the groups rank the attributes, no statistical analysis is performed. On the contrary, the Friedman test can be applied when the ranking task is performed individually. This fact is essential, as these results would provide crucial information on the critical attributes for consumer acceptance.

If the acceptance test is included, ANOVA and Tukey tests are performed (Auriema et al. 2021; da Costa et al. 2020; Grygorczyk et al. 2013; McSweeney et al. 2016; J. M. da Silva et al. 2021). Finally, when groups of consumers or PAE sessions are compared, MFA can be performed (Auriema et al. 2021; da Costa et al. 2020; Grygorczyk et al. 2013; McSweeney et al. 2016; J. M. da Silva et al. 2021). This method allows us to visualize the relationship between the sensory profile obtained by the PAE method according to the different groups of consumers (J. M. da Silva et al. 2021). RV coefficient (0–1) may be used to analyze the importance of the correlation, and the higher this number, the higher the correlation between the configurations (da Costa et al. 2020; McSweeney et al. 2016; J. M. da Silva et al. 2021).

In the 22 studies reviewed, the number of attributes evaluated using the PAE method ranged from 5 to 23 (13.07 ± 3.98), with most studies reporting between 10 and 20 attributes. The panels were predominantly composed of frequent consumers of the target products (17.09 ± 8.96), often recruited based on a minimum consumption frequency. At the same time, some studies included specific groups such as vegans, vegetarians, or consumers with prior exposure to the product or related processing technologies. In some instances, participant groups were segmented by gender or previous experience with sensory or quality evaluation, allowing for comparative analyses (e.g., Popoola et al. 2019; Muggah et al., 2017). Furthermore, the number of samples was from 1 to 10 (4.23 ± 1.80). Regarding training, most studies have not reported using formal sensory training, which aligns with the objective of PAE, which is to capture consumers’ spontaneous vocabulary and perception. However, some studies described a brief familiarization phase with the procedure, mainly during the initial attribute elicitation and scale development steps, aimed at facilitating participants’ understanding of the task (e.g., T. O. Silva et al. 2024; Grygorczyk et al. 2013). Additional information, such as the number and type of panelists, the statistical approach used, and key findings reported by the authors, can be found in Table 1.

**TABLE 1:** Preferred attribute elicitation (PAE) applied in the descriptive sensory analysis of food products.

Product	Assessors type and number of attributes and samples	Statistical approach and duration	Objective of PAE	Results using PAE	Reference
**Fruit‐based products**					
Mangaba pulp fermented with probiotics	Number of participants = 11 Consumers of fruits and derivatives Number of attributes = 20 Number of samples = 3 in 2 storage times	GPA Friedman Test 120 min	Evaluate the impact of probiotic fermentation on the sensory attributes during storage (7°C, 28 days)	Differences in the sensory attributes between probiotic and conventional mangaba pulps, with improved characteristics for the probiotic products Differences in the sensory attributes during refrigerated storage in few attributes	de Assis et al. (2024)
Mango and melon with edible coatings with probiotics and/or prebiotics	Number of participants = 10 Consumers of fruits Number of attributes = 20 Number of samples = 4	GPA 120 min	Evaluate the impact of edible coatings on the sensory attributes during storage (5 ^o^C, 12 days)	Differences in the sensory attributes according to the edible coating type and fruit (mango or melon) Differences in the sensory attributes during refrigerated storage	Dias et al. (2023)
Melon and papaya with citral nanoemulson	Number of participants = 8 Consumers of fruits Number of attributes = 11 Number of samples = 4 in 2 storage days	GPA 120 min	Evaluate the impact of citral nanoemulsion on the sensory attributes during storage (4°C, 7 days)	Differences in the sensory attributes between fruits with or without citral nanoemulsion, with improved characteristics for the fruits with citral nanoemulsion No impact of storage on sensory characteristics	Luciano et al. (2023)
**Meat products**					
Chicken mortadella with green banana biomass	Number of participants = 27 (group1 with 14 and group 2 with 13) Consumers of mortadella (at least once a week) Number of attributes = 10‐15 depending on the PAE session Number of samples = 5	GPA MFA (for different PAE sessions) 90 min	Evaluate the impact of green banana biomass on the sensory attributes and differences on the results for different PAE sessions	Differences in the sensory attribute's intensity according to the concentration of green banana biomass used, with better results for 75% Slight changes in the sensory attribute's intensity according to the PAE session	Auriema et al. (2021)
Beef, elk, horse, and bison meat	Number of participants = 7 to 11 depending on the PAE session Meat consumers with or without sensory evaluation and meat quality experience depending on the PAE session Number of attributes = 7–11 depending on the PAE session Number of samples = 4	GPA MFA Session duration not mentioned	Characterize the sensory characteristics of beef, elk, horse, and bison meats	Differences in the sensory attribute's intensity according to the meat type Similar results to CATA (RV = 0.843–0.925) Sensory evaluation and meat quality experience are important attributes to defining panel size	Popoola et al. (2019)
**Dairy products**					
Goat milk yogurt with probiotics and/or xique‐xique jam	Number of participants = 5 Consumers of goat milk yogurt that have already consumed xique‐xique in foods Number of attributes = 16 Number of samples = 3	GPA 75 min for the first session and 25 min for the second and third sessions	Evaluate the impact of xique‐xique jam and/or probiotic culture on the sensory attributes during storage (5°C, 28 days)	Probiotic and/or xique‐xique addition impacted positively the sensory attributes intensity Determination of the most adequate storage time for maintaining the sensory characteristics of the products (14 days)	Bezerril et al. (2022)
Goat milk fermented milk with probiotics and/or prebiotics	Number of participants = 30 (group 1 with 15 and group 2 with 15) Consumers of fermented milk (at least once a week) Number of attributes = 9 Number of samples = 3	GPA ANOVA and Tukey (for acceptance) 70 min	Evaluate the impact of block freeze concentration and probiotic and/or prebiotic addition on the sensory attributes	Block freeze concentration and probiotic and/or prebiotic addition impacted the sensory attributes intensity Block freeze concentration impacted negatively on the sensory properties No impact of gender on products characterization	Canella et al. (2024)
Probiotic yogurts with different sweeteners	Number of participants = 20 Consumers of yogurt (at least once a week) Number of attributes = 10 Number of samples = 5	GPA Friedman test ANOVA and Tukey (for acceptance) MFA (for DA and PAE data) 75 min	Assess the impact of different sweeteners or prebiotics on the sensory characteristics of probiotic yogurts, compare to conventional descriptive analysis (DA), and study the sensory acceptance compared to external consumers	Differences in the sensory attribute's intensity according to the type of sweetener High similarity between PAE and DA configurations (RV = 0.92, *p* = 0.02) Similar acceptance scores for PAE consumers and external consumers	Costa et al. (2020)
Yogurt with different textures	Number of participants = 42 (10 for each session) Consumers of yogurt (at least once a week) Number of attributes = 5–11 depending on PAE session Number of samples = 7	GPA ANOVA and Tukey (for acceptance) MFA (for DA and PAE data) 70–90 min	Assess the impact of different textures on the texture characteristics of yogurts, compare to conventional DA	High similarity between PAE and DA configurations (RV = 0.877, *p* = 0.0011) Similar acceptance scores for PAE consumers and external consumers Similar configurations for different gender sessions	Grygorczyk et al. (2013)
Dulce de leche processed by ohmic heating	Number of participants = 20 Type of consumer not stated Number of attributes = 9 Number of samples = 5	GPA ANOVA and Tukey (for acceptance) MFA (for DA and PAE data) HCA 70 min	Assess the impact of ohmic heating on the sensory characteristics	Differences in the sensory attribute's intensity according to the ohmic heating application and processing parameters, with better results for 8 or 10 V/cm	R. Silva et al. (2020)
Coalho cheese	Number of participants = 20 Consumers of Coalho cheese (at least once a week) Number of attributes = 13–23 depending on the region of the country Number of samples = 10	GPA MFA (for data of different regions) Bootstrapping resampling approach 60–70 min	Assess the impact of country region on the sensory characteristics	Differences in the sensory attribute's intensity according to the country region, with better discrimination for the south region Recommendation of a minimum number of participants of 19	Soares et al. (2019)
Goat ricotta cream with probiotics	Number of participants = not described Consumer type = not described Number of attributes = 13–23 depending on the region of the country Number of samples = 3 in 3 storage days	PCA Session duration not mentioned	Assess the impact of *Limosilactobacillus mucosae* on the sensory characteristics during storage (4°C, 14 days)	Differences in the sensory attribute's intensity according to the probiotic addition, with better results for the probiotic product	Viana et al. (2024)
**Dairy alternatives**					
Probiotic Baru almond beverage added with prebiotics	Number of participants = 15 Consumers of yogurt, fermented milk, dairy beverages or soy beverages (daily) Number of attributes = 13 Number of samples = 5	GPA Friedman test ANOVA and Tukey (for acceptance) 120 min	Assess the impact of different prebiotics on the sensory characteristics of probiotic fermented beverages	Differences in the sensory attribute's intensity according to the type of prebiotics	Fernandes et al. (2021)
Rice by‐product frozen desserts added with prebiotics	Number of participants = 22 for each group (vegans and nonvegans) Consumers according to the diet type Number of attributes = 13 for nonvegans and 17 for vegans Number of samples = 5	GPA Friedman test MFA (for vegan and nonvegan data) 120 min	Assess the impact of different prebiotics on the sensory characteristics of frozen desserts and study the differences in characterization between vegan and nonvegan consumers	Differences in the sensory attribute's intensity according to the type of prebiotics Agreement between vegan and nonvegan consumers	J. M. da Silva et al. (2021)
Rice by‐product frozen desserts added with prebiotics, probiotics and *Spirulina platensis*	Number of participants = 15 Consumers of ice cream and plant‐based products Number of attributes = 8 Number of samples = 4 in 2 storage days	GPA Friedman test ANOVA and Tukey (for acceptance) 120 min	Assess the impact of probiotic and prebiotic addition on the sensory characteristics of frozen desserts during storage (–18°C, 120 days)	Differences in the sensory attributes between probiotic and conventional frozen desserts, with improved characteristics for probiotic products Stability during storage	de Souza et al. (2023)
Cashew nut extract processed by ultrasound	Number of participants = 5 Consumers of vegetable milks (> 3 days a week) Number of attributes = 12 Number of samples = 2 in duplicates	GPA Friedman test ANOVA and Tukey (for acceptance) Session duration not mentioned	Assess the impact of ultrasound on the sensory characteristics of cashew nut extracts	Differences in the sensory attributes between ultrasound and pasteurized products, with improved characteristics for the ultrasound‐treated product Stability of configurations for duplicate samples	da Silveira Maia et al. (2025)
Baru almond fermented beverages processed by ultrasound	Number of participants = 15 Consumers of nondairy fermented beverages (> 3 days a week), including one vegan and two vegetarians Number of attributes = 14 Number of samples = 3 in 2 storage days	GPA Friedman test ANOVA and Tukey (for acceptance) Session duration not mentioned	Assess the impact of ultrasound on the sensory characteristics of Baru almond fermented beverages	Ultrasound impacted positively on the sensory attributes when probiotic was added before it	Rocha et al. (2023)
**Bakery products**					
Millet‐based snacks and biscuits	Number of participants = 24–25 each session Consumers of biscuits and extruded snacks (> once a month) Number of attributes = 9 Number of samples = 4	GPA ANOVA and Tukey (for acceptance) MFA (for male and female data) 60–90 min	Evaluate the impact of millet concentration on the sensory attributes	Differences in the sensory attribute's intensity according to the concentration of millet used, with better results for 25% Similar acceptance scores for PAE consumers and external consumers	McSweeney et al. (2016)
Cookies with green tea extract	Number of participants = 18 or 25 depending on PAE session Consumers of cookies (> once every 2 weeks) Number of attributes = 8–10 depending on the PAE session Number of samples = 5	GPA ANOVA and Tukey (for acceptance) 75 min	Evaluate the impact of green tea extract concentration on the sensory attributes	Low similarity between PAE and DA configurations (RV = 0.179–0.554) High similarity between PAE sessions (RV = 0.843)	McSweeney et al. (2017)
**Beverages**					
Beer	Number of participants = 11–17 depending on the PAE session (males or females) Regular consumers of beer (consumption in the last 2 weeks) Number of attributes = 11–17 depending on the PAE session Number of samples = 4	GPA Session duration not mentioned	Evaluate the impact of gender on the sensory attributes	Males and females evaluated the beers differently (RV = 0.117–0.196) No significant differences between PAE sessions for the same gender (RV = 0.991–0.998)	Muggah et al. (2017)
Ginger beer	Number of participants = 11 Consumers of beer and ginger‐based products Number of attributes = 17 Number of samples = 1 in 3 fermentation times	GPA Friedman 120 min	Evaluate the impact of fermentation time on the sensory attributes	Differences in the sensory attributes of ginger beers during storage, with improved characteristics for the 14 days of storage compared to 7 days	de Oliveira et al. (2024)
Probiotic kombucha	Number of participants = 14 Consumers of kombucha beverages Number of attributes = 16 Number of samples = 4	GPA Friedman Session duration not mentioned	Evaluate the impact of probiotic and/or camu‐camu pulp addition on the sensory attributes	Differences in the sensory attribute's intensity according to the addition of probiotics and/or camu‐camu pulp, with better results for both ingredients	T. O. Silva et al. (2024)

ANOVA: analysis of variance; CATA: check all that apply; DA: descriptive analysis; GPA: generalized procrustes analysis; HCA: hierarchical cluster analysis; MFA: multiple factor analysis; PAE: preferred attribute elicitation.

### 4.2 PAE in the DA of Food Products

**FIGURE 3:**
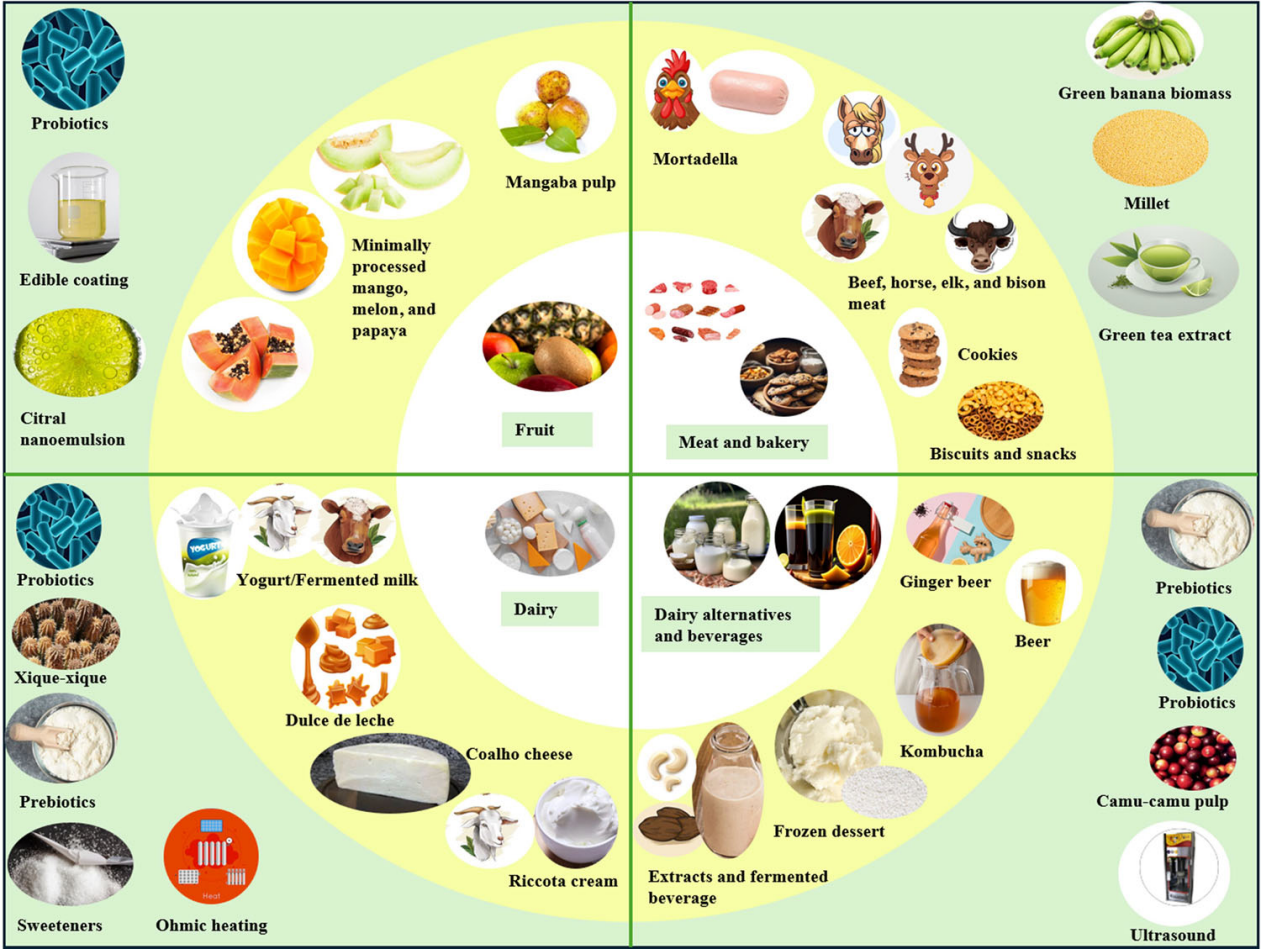
Main applications of preferred attribute elicitation (PAE) in the sensory descriptive analysis of food products.

Figure 3 presents the main applications of PAE in the sensory DA of food products. The PAE method has been applied for evaluating the sensory attributes of several products, such as fruits and derived products (de Assis et al. 2024; Dias et al. 2023; Luciano et al. 2023), meat products (Auriema et al. 2021; Popoola et al. 2019), dairy products (Bezerril et al. 2022; Canella et al. 2024; da Costa et al. 2020; Grygorczyk et al. 2013; R. Silva et al. 2020; Soares et al. 2020; Viana et al. 2024), dairy alternatives (Fernandes et al. 2021; da Silveira Maia et al. 2025; Rocha et al. 2023; J. M. da Silva et al. 2021; de Souza et al. 2023), bakery products (McSweeney et al. 2016, 2017), and beverages (Muggah and McSweeney 2017; de Oliveira et al. 2024; T. O. Silva et al. 2024).

Table 1 presents the primary studies with PAE application in the sensory DA of food products. The application of PAE allowed the evaluation of the impact of processing techniques on the intensity of sensory attributes in studies with fermentation using probiotic cultures (Bezerril et al. 2022; de Assis et al. 2024) or the application of emerging technologies (R. Silva et al. 2020; Rocha et al. 2023; Canella et al. 2024; da Silveira Maia et al. 2025).

The PAE method reported the positive impact of probiotic fermentation on the sensory attributes of mangaba pulp (de Assis et al. 2024), yogurt (Bezerril et al. 2022), and kombucha with camu camu pulp (T. O. Silva et al. 2024). Despite the absence of prior training, participants generated an extensive list of attributes in these studies. PAE method discriminated the samples based on the sensory attributes. The probiotic products presented improved sensory characteristics, such as mangaba pulp with higher intensity of yellow color, brightness, fresh appearance, and typical aroma and flavor (de Assis et al. 2024), goat milk yogurt with stronger herbaceous flavor and aroma, sweet aroma and taste, dairy aroma, and typical aroma and flavor (Bezerril et al. 2022), and fruity kombuchas with higher intensity of fruity flavor, yellow color, aerated appearance, and citric flavor (T. O. Silva et al. 2024) compared to the traditional (without probiotic) products.

Meanwhile, PAE also reported improved sensory characteristics for products processed by emerging technologies compared to pasteurized products, such as cashew nut extracts or Baru almond fermented beverages processed by ultrasound (Rocha et al. 2023; da Silveira Maia et al. 2025) and dulce de leche processed by ohmic heating (R. Silva et al. 2020). In these cases, the PAE method discriminated the samples based on the sensory attributes, and the products subjected to emerging technologies showed better sensory properties, such as ultrasound‐treated cashew nut extracts with higher consistency and lower presence of granules (da Silveira Maia et al. 2025), ultrasound‐treated fermented beverages with higher intensity of sweet aroma/taste and almond flavor (Rocha et al. 2023) and ohmic heating‐treated dulce de leche with higher brightness, sweet taste, dulce de leche aroma and flavor, and fluidity (R. Silva et al. 2020) than the pasteurized products. On the contrary, it reported that the freeze concentration of goat milk would not be a good option for fermented milk processing, resulting in products with undesirable sensory characteristics (goat flavor, acid taste, and salty taste) (Canella et al. 2024).

Therefore, this technique distinguishes products processed with innovative methods, such as ultrasound and ohmic heating, from conventional processing (pasteurization). In addition, the methodology revealed that consumers identified sensory characteristics similar to those described in previous descriptive analyses, reinforcing the convergent validity of PAE for sensory characterization of products processed through emerging technologies. Finally, PAE also assessed the enhanced sensory characteristics in mango and melon coated with edible coatings, particularly those containing probiotics and prebiotics (synbiotic coatings) (Dias et al. 2023). The fruits with synbiotic coatings presented improved sensory properties, such as firm texture, smooth texture, brightness, and moist appearance (Dias et al. 2023).

Notably, the sensory data of the DA could be related to the physicochemical properties, such as volatile compounds, organic acids, and sugar contents, color and texture parameters (de Assis et al. 2024; Bezerril et al. 2022; Dias et al. 2023; da Silveira Maia et al. 2025; T. O. Silva et al. 2024) or sensory acceptance (Canella et al. 2024; da Silveira Maia et al. 2025; T. O. Silva et al. 2024). These results state this method's importance in assessing the effects of processing techniques on the sensory attributes of food products and their correlation with physicochemical analysis and sensory acceptance. Also, they allow us to study possible synergistic effects of ingredients by comparing the sensory characteristics of food products with isolated or combined ingredients (such as the synergistic effect of prebiotics and probiotics in the edible coatings, Dias et al. (2023) or probiotics and camu‐camu pulp in kombucha (T. O. Silva et al. 2024). Furthermore, it could state the most suitable processing parameters for emerging technologies (8 or 10 V/m for ohmic heating in dulce de leche (R. Silva et al. 2020), aiming for products with improved sensory characteristics. In this case, PAE allowed distinguishing products processed under different electric field intensities, associating higher brightness and fluidity with higher intensities. In contrast, intermediate intensities increased bitterness and reduced sweetness (R. Silva et al. 2020).

The application of PAE also allowed the evaluation of the impact of ingredients on the intensity of sensory attributes, such as green banana biomass (fat replacer) in chicken mortadella (Auriema et al. 2021), natural sweeteners and/or prebiotics (sugar replacer) in yogurts (da Costa et al. 2020), prebiotics to Baru almond fermented beverages (Fernandes et al. 2021) or rice frozen dessert (J. M. da Silva et al. 2021), citral nanoemulsion to melon and papaya (Luciano et al. 2023), millet in biscuits and snacks (McSweeney et al. 2016), green tea extract in cookies (McSweeney et al. 2017), and probiotics in frozen dessert of rice byproduct (de Souza et al. 2023) or goat ricotta cream (Viana et al. 2024). In these studies, the PAE method noted that citral nanoemulsion and probiotics contributed to improving the sensory characteristics of fruits (Luciano et al. 2023), frozen desserts (de Souza et al. 2023) and goat ricotta cream (Viana et al. 2024). Therefore, the citral nanoemulsion use resulted in melon and papaya with higher intensities of brightness, typical appearance, succulence, firmness, and herbal aroma and flavor (Luciano et al. 2023), while probiotics contributed to increased mint aroma and flavor intensity in frozen desserts (de Souza et al. 2023), and fermented and acid taste in goat ricotta cream (Viana et al. 2024).

Furthermore, products with different sensory characteristics were obtained depending on the concentration of fat replacers in chicken mortadella or type of sweetener and prebiotic in yogurts, nondairy fermented beverages, and nondairy frozen desserts (Auriema et al. 2021; da Costa et al. 2020; Fernandes et al. 2021; J. M. da Silva et al. 2021), which may allow industries to customize products according to specific market niches. In addition, it reported that millet and green tea extract negatively impacted the sensory properties of biscuits and snacks (McSweeney et al. 2016) and cookies (McSweeney et al. 2017), respectively. PAE analysis also allows to define the optimum concentration of ingredients or ingredient types aiming to maintain or improve the sensory attributes considered important for sensory acceptance by consumers, such as 75% for green banana biomass in chicken mortadella (Auriema et al. 2021), 25% of millet in biscuits and snacks (McSweeney et al. 2016) or 4% of green tea extract in cookies (McSweeney et al. 2017). Therefore, the results report the importance of the PAE method in reformulating products using functional and natural ingredients, aligning with a growing consumer demand for healthier foods.

The PAE method has also been used for determining the impact of storage on the sensory attributes of food products, such as mangaba pulps fermented with probiotic cultures (de Assis et al. 2024), yogurts fermented with probiotic cultures and xique‐xique jam (Bezerril et al. 2022), minimally processed fruits with edible coatings (Dias et al. 2023), fruits with citral nanoemulsion (Luciano et al. 2023), and frozen desserts with probiotics (de Souza et al. 2023). At the same time, it was used to evaluate fermentation time's impact on ginger beer's sensory attributes (de Oliveira et al. 2024). The results of PAE application during storage time state its applicability to note the attributes that were most impacted by storage time (de Assis et al. 2024; Dias et al. 2023) and to state the best shelf life for the products based on the improved sensory characteristics (Bezerril et al. 2022). In other cases, it reports maintenance of the sensory characteristics during the evaluated shelf life (Luciano et al. 2023; de Souza et al. 2023). This approach is interesting, as shelf life is often established by considering only microbiological characteristics, increasing the number of consumers complaining about the sensory characteristics of the products at the end of storage time. Finally, it reported the positive impact of fermentation on the characteristics of ginger beers, with 14 days providing better sensory characteristics than 7 days (de Oliveira et al. 2024). This way, the fermentation time may also be optimized based on the sensory characteristics of the products.

In addition, the PAE method may be used to differentiate commercial food types, such as macro brewed, crafted, IPA and lager beers (Muggah and McSweeney 2017), beef, elk, horse, and bison meats (Popoola et al. 2019) or Coalho cheeses with different fat and lactose contents (Soares et al. 2020) based on their sensory characteristics. In these cases, the PAE method denoted the sensory attributes that characterized each brewer, beer type, and meat and cheese type. These results suggest that future studies could use the PAE method to verify the authenticity of food products, considering their typical sensory properties.

Finally, the PAE method has also been used for comparisons between the perception of food products using different groups of consumers (Grygorczyk et al. 2013; Muggah and McSweeney 2017; J. M. da Silva et al. 2021; Soares et al. 2020). In this way, differences in the characterization of food products were evaluated based on gender for beers (Muggah and McSweeney 2017) and yogurts (Canella et al. 2024; Grygorczyk et al. 2013), consumers from different regions of the same country for Coalho cheese (Soares et al. 2020), or vegan and nonvegan consumers for rice frozen dessert (J. M. da Silva et al. 2021). The results allow us to note that although the elicited sensory attributes may be similar between groups, their importance for consumer acceptance and/or product characterization can differ between vegan and nonvegan consumers (J. M. da Silva et al. 2021), male and females (Muggah and McSweeney 2017), and participants from different regions of the same country (Soares et al. 2020). However, gender differences have not been denoted in other studies (Canella et al. 2024; Grygorczyk et al. 2013). The evaluation of different groups of consumers is important for tracing marketing strategies and evaluating the perceptions of each group type.

PAE offers several advantages over DA and other rapid sensory methods, including its application in a single session (in most cases), the use of consumers as assessors, the identification of key attributes for product acceptance, and the quantification of sensory attribute intensities in the products (Grygorczyk et al. 2013). This method typically generates fewer attributes than other rapid sensory descriptive methods due to the consensus among participants, which reduces variability in panelists' definitions of attributes (McSweeney et al. 2017).

As a general observation of the studies using the PAE, a higher number of studies show the utilization of nonbovine milk (mainly goat milk) and nonbovine meat (horse, bison, and elk), utilization of emerging technologies (ultrasound or ohmic heating), development of dairy alternatives, the inclusion of functional ingredients (probiotics and prebiotics) to healthy products (fermented milk, yogurt, fruits, fruit pulps, and kombucha), utilization of nonconventional ingredients (Cactaceae, underexplored grains and nuts, byproducts, green tea extract, and microalgae), and fat and sugar replacement. This utilization may be related to the higher number of studies in these areas in the last years. Therefore, there are opportunities for studies to consider other food product categories, including the more conventional products, if the researchers find suitability in PAE application.

## 5 General Overview, Factors, Strengths, Limitations, and Directions

**FIGURE 4:**
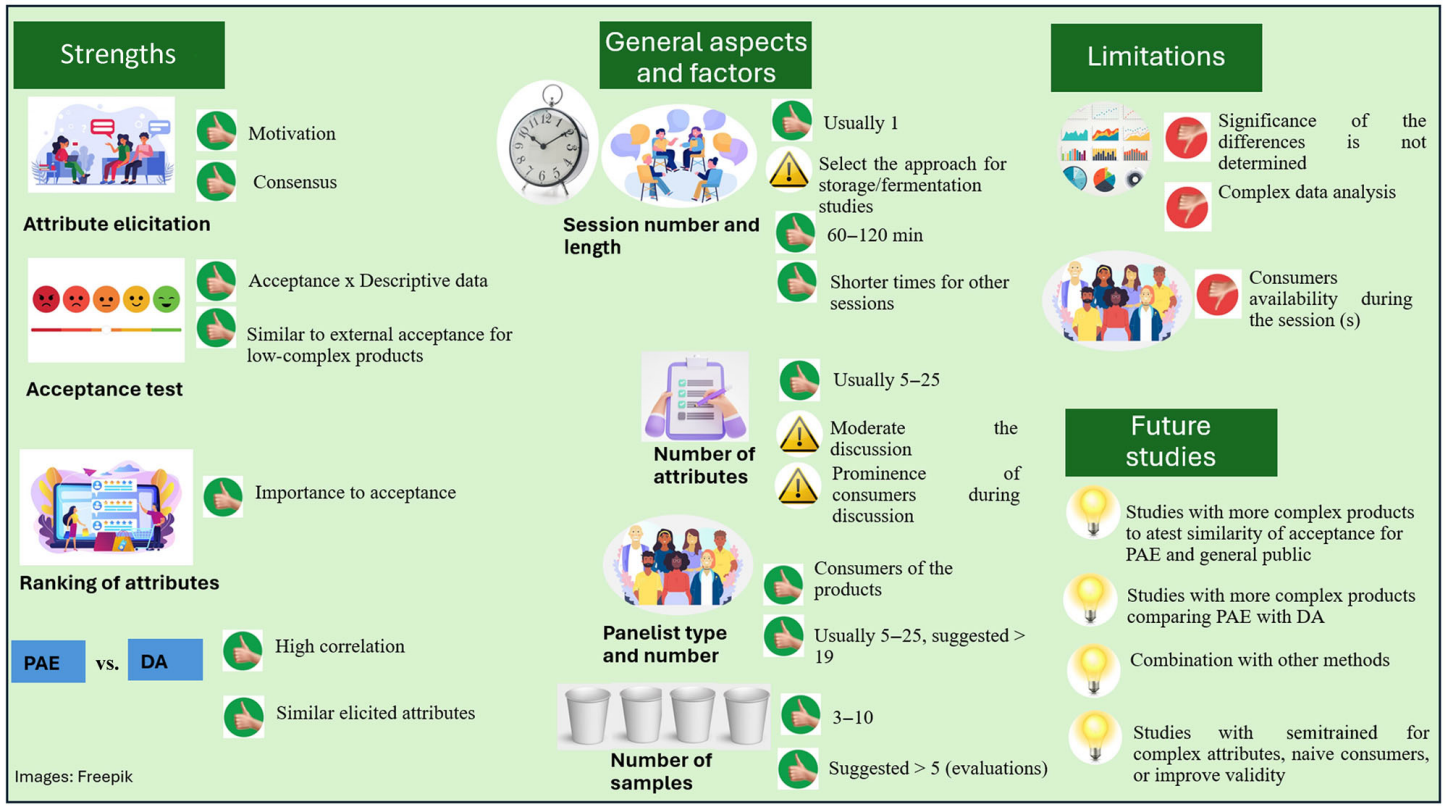
General overview, factors, strengths, limitations, and directions of PAE application in food products.

Figure 4 presents the general overview, factors, strengths, limitations, and directions of PAE application in food products. PAE method has some strengths. The attribute elicitation step has been considered one differential of PAE from other rapid sensory methods, such as CATA, JAR, and ideal profiling, which commonly use predefined terms. In this way, in PAE, the attributes are selected by the group's consensus, similar to the DA method.

Furthermore, the PAE group is also used to evaluate the acceptance of the products. This way, PAE utilization may provide insights and explanations on higher or lower sensory acceptance (Canella et al. 2024). A key question is whether the acceptance supplied by a small number of consumers would correspond to that of a higher number of consumers. Previous studies compared the acceptance with PAE participants (*n* = 20–25) and general consumers (*n* = 65–90) for yogurts, biscuits, and snacks, and no differences were noted (da Costa et al. 2020; Grygorczyk et al. 2013; McSweeney et al. 2016). It is important to point out that the products evaluated in the studies had low complexity, were familiar to the consumers, and presented slight differences in their attributes. Therefore, the results should be considered cautiously, and the good performance would be mainly for low‐complexity products and early product development evaluation. More studies with more complex products are recommended, highlighting opportunities to explore this topic.

In addition, the PAE method allows for ranking attributes according to their importance for sensory acceptance, considering the consumer's point of view. Therefore, it is possible to note the important attributes and highlight the undesired ones, allowing the industries to perform resamples based on consumer perception. It also can help product developers focus on the most critical attributes to drive consumer liking and gain a competitive advantage in the market, as differences in the intensity of sensory attributes with a low impact on consumer acceptance may not need any industry action. In contrast, the changes in attributes that are highly important for consumer acceptance may be carefully evaluated.

This way, the PAE method has provided important results for the sensory characterization of food products. GPA maps with a high explained variation in the first components (> 60%) have been observed, which is considered relevant for studies with nontrained panelists, as they may have more difficulties than trained panelists in performing the task (de Assis et al. 2024; Auriema et al. 2021; Dias et al. 2023; Popoola et al. 2019; J. M. da Silva et al. 2021). In some cases, explanations higher than 90% were achieved, which are similar values to those found in DA (Bezerril et al. 2022; Canella et al. 2024; da Costa et al. 2020; da Silveira Maia et al. 2025; de Oliveira et al. 2024). Using untrained participants may save time and resources compared to methods using trained participants, such as DA (Canella et al. 2024). Some approaches have been performed to verify the performance of the PAE method, such as performing different sessions with different participants (Auriema et al. 2021; Muggah and McSweeney 2017) and genders (Grygorczyk et al. 2013; McSweeney et al. 2016, 2017), or including duplicate samples for evaluation (da Silveira Maia et al. 2025). A high correlation between sessions or product configurations was observed, demonstrating agreement on the assessment and the method's robustness. Therefore, the products were differentiated in the same way, but not necessarily by the same attributes.

Comparisons between PAE and DA have been performed, which is of utmost importance, considering that DA is a consolidated reference method (da Costa et al. 2020; Grygorczyk et al. 2013; McSweeney et al. 2017). The attributes elicited in both methods were similar, and they provided identical configurations for yogurt texture (Grygorczyk et al. 2013, RV = 0.877) and yogurt general overall attributes (da Costa et al. 2020; RV = 0.92), while no significant correlation between DA and PAE configurations was observed for cookies (McSweeney et al. 2017). A previous study also reported a similarity between PAE and CATA configurations, one of the most used rapid sensory methods (Popoola et al. 2019, RV = 0.843–0.925). In this way, PAE may be recommended as an alternative for DA when a rapid characterization of the products is needed, and the perception of potential buyers (consumers) is advisable. In the case of cookies, the lack of correlation could not be explained in the study; however, it is important to note that the authors performed modifications of the methodology, which may have impacted its performance. We reinforce the need for more studies comparing PAE with DA and other sensory methods to better understand its strengths and limitations. Although DA and PAE have a discussion phase to select the attributes, potential biases may appear due to differences in trained assessors and consumer forms of selecting and evaluating the attributes.

One interesting approach is to use PAE with another sensory method to complement the results and better understand the sensory characteristics of the products. This initiative has been performed by aligning PAE with TCATA in dulce de leche processed by ohmic heating, where PAE provided a complete characterization of the samples and TCATA, which is a dynamic method, reported the changes and evolution of the attributes over evaluation time (R. Silva et al. 2020). At the same time, JAR and Ideal Scale test was used with PAE to characterize goat ricotta cream with *Limosilactobacillus mucosae* CNPC007 or *Lactobacillus acidophilus*, where PAE provided information about the changes in the attributes after probiotic addition and JAR and Ideal scale stated the attributes lower, ideal, or greater than the ideal according to consumer perceptions (Viana et al. 2024). The combinations of methods, in these cases, are important, as static methods (like PAE) may not provide information on residual sweetness and bitterness or the ideal products according to the attribute intensity. It is important to highlight that it is possible to ask the consumers to evaluate the intensity of the attributes at different times of evaluation (beginning, middle, or end) using PAE. Therefore, further studies could evaluate the applicability of PAE in these circumstances, similar to what occurred to CATA and TCATA. By combining different methods, the food industry can obtain a more comprehensive knowledge of the sensory attributes of the products, make informed decisions concerning product development, quality control, and marketing, and get some insights on the temporal aspects of sensory perception (Rodrigues et al. 2024). However, it is important to note that PAE has not been used in dynamic tests at the moment.

Considering the general information on the application of the method, usually, the PAE method was applied only in one session (de Assis et al. 2024; Auriema et al. 2021; Canella et al. 2024; da Costa et al. 2020; Fernandes et al. 2021). However, more sessions may be needed in studies using different storage or fermentation times. Two approaches may be used in these cases, and the selection should consider data visualization on the GPA map. The first approach is to perform the PAE method in one session using all the samples (from all storage or fermentation times) (de Assis et al. 2024; Luciano et al. 2023; de Oliveira et al. 2024; de Souza et al. 2023). In this case, it is possible to include all samples and storage/fermentation times in the same GPA map (Figure 2B), allowing the visualization of the storage/fermentation impact on the sensory characteristics of the products. However, it is important to note that to have products with different storage/fermentation times simultaneously, they will not be from identical batches. This would be problematic for products with high variation in sensory attributes in different lots.

The second approach is to perform the PAE method with various sessions, one in each storage/fermentation interval (Bezerril et al. 2022; Dias et al. 2023). In this case, including all samples and storage/fermentation times in the same GPA map is not recommended. Therefore, one GPA map is constructed for each storage/fermentation time (Figure 2C), and the storage/fermentation impact on the sensory characteristics of the products is not visualized directly. In this approach, the products could be from the same lot and evaluated effectively during storage/fermentation time. Finally, in studies involving the comparison of sensory profiles between groups (vegan and nonvegans, different genders or regions of the country), one session is performed with each group (Auriema et al. 2021; Grygorczyk et al. 2013; McSweeney et al. 2017; Muggah and McSweeney 2017; J. M. da Silva et al. 2021; Soares et al. 2020).

The sessions have lasted 60 min (McSweeney et al. 2016), 70 min (R. Silva et al. 2020), 75 min (Bezerril et al. 2022; da Costa et al. 2020; Muggah and McSweeney 2017), 90 min (Auriema et al. 2021), or 120 min (de Assis et al. 2024; Dias et al. 2023; Fernandes et al. 2021; Luciano et al. 2023; de Oliveira et al. 2024; J. M. da Silva et al. 2021; de Souza et al. 2023). For the studies with more than one session, a shorter period (25 min) was needed from the second session (Bezerril et al. 2022), as steps 3–5 are not required. We could observe that, generally, shorter session times occurred for products of overall consumption and high familiarization by consumers (such as yogurts, mortadella, biscuits, and snacks). Longer sessions were observed for nonconventional products (such as nondairy fermented beverages and frozen desserts, probiotic fruit pulps, fruits with edible coatings or citral nanoemulsions, and ginger beers). Therefore, the researchers should consider the food product type to estimate the session length. This estimation is important in recruiting participants to check their availability to participate.

The number of attributes is an important factor for the performance of the PAE method. Previous studies have reported differences in the number of elicited attributes, ranging from 5 (Grygorczyk et al. 2013) to 25 (McSweeney et al. 2016, 2017). The number of attributes depends on the evaluated product, the participants, and the attribute category evaluated. Studies evaluating only one attribute type (texture, for example) (Grygorczyk et al. 2013) may have fewer attributes. Too few attributes may encourage similar responses for all samples, while too many attributes may result in fatigue. Moderator discussions with the panelists in the “attribute definition” step are crucial to reducing the number of attributes and keeping the important ones.

The panelist type is also an important factor to be considered. Generally, consumers of the studied products have been used as panelists, and the researchers defined the minimum consumption of the product for their eligibility. Furthermore, availability for at least 1 h on an established day (da Costa et al. 2020) and not being an employee of the food industry (McSweeney et al. 2016, 2017; Muggah and McSweeney 2017) are commonly requested. Selecting consumers as panelists seems to be an established approach, but the minimum product consumption depends on the study's objectives and the participant's availability. However, it is essential to note that, in some cases, it is challenging to obtain participants who are consumers of the products (such as for the case of elk, horse, and bison meats) and consumers of similar products (beef) could be used (Popoola et al. 2019).

Although all the studies with PAE used consumers as participants, we highlight here that semitrained participants may be used when some attributes of the products are complex, if the participants have difficulties using the scale, or to improve the validity and repeatability when using few participants. In this case, the participants are subjected to a short training course, which may treat inconsistencies (Rodrigues et al. 2024).

In addition, the number of panelists is another important factor. Previous studies have used different numbers of participants, ranging from 5 (da Silveira Maia et al. 2025) to 25 (McSweeney et al. 2017). The selection of the number of participants may consider the knowledge/familiarization about the food product, recommending a more significant size panel for completely naïve participants. In contrast, lower‐size panels may be used for participants with product or sensory evaluation experience (Popoola et al. 2019). We could observe that, in the studies considering exotic or innovative products, the authors preferred to apply the method with fewer participants who were effective consumers of the products (de Assis et al. 2024; Bezerril et al. 2022).

A previous study used the bootstrapping resampling approach and RV to estimate the minimum number of participants for the PAE method. They suggested a minimum of 19 participants to obtain a stable sample configuration (Soares et al. 2020). This minimum number was considered in studies in the literature (da Costa et al. 2020; J. M. da Silva et al. 2021; R. Silva et al. 2020). Based on our experience with this method, using at least 19 consumers is interesting, as this number has already been validated in an intracultural study. However, it is sometimes difficult to find this number of consumers for some food types, and a lower number of consumers may be used. Overall, it is crucial to clarify how the number of consumers in studies is determined to avoid misunderstandings in reproducing PAE. Furthermore, it is important to mention that the minimum number of participants proposed by Soares et al. (2020) was established using the descriptive data, but it could be low for the acceptance test, and the PAE performance may be product‐dependent.

Finally, the number of samples should be considered. The PAE studies evaluated from 3 (Bezerril et al. 2022; Canella et al. 2024) to 10 samples (Soares et al. 2020). We could observe that most of the studies assessed the sensory profile of five different samples (Auriema et al. 2021; da Costa et al. 2020; Fernandes et al. 2021; McSweeney et al. 2017; J. M. da Silva et al. 2021; R. Silva et al. 2020). It is recommended to include at least five to six samples to allow assessment of the correlation between sensory attributes in the GPA map. However, it is essential to note that, in theory, the minimum number of samples to calculate a correlation is three. In this case, however, the correlations may be overestimated, and the data may not be enough to characterize the sensory space (Hasted 2017). In some cases, a total of five evaluations (not samples) were made, that is, fewer samples at different storage times (Luciano et al. 2023; Rocha et al. 2023; de Souza et al. 2023; de Assis et al. 2024; Viana et al. 2024), which could be considered sufficient for constructing the GPA map. We highlight here that the number of samples should be stated considering the product type, with a reduced number for samples with an intense and persistent flavor. Using at least five samples/evaluations may comply with statistical recommendations. However, the number of samples should be chosen based on the study's objective and product type.

However, the PAE method may have some limitations, considering previous studies. First, different sessions for the same product may result in differences in the characterization of food products, which may be associated with wrong interpretation by the participants or problems in the attribute evaluation form (Auriema et al. 2021). Therefore, steps 3 and 4 are of utmost importance for the performance of the PAE method, and the moderator should provide a discussion with the group on the suitable attributes to be evaluated, the scale, and the form of assessing each attribute. Attributes that are generally recognized and have a clear concept usually do not present problems for evaluation. On the other hand, when multidimensional attributes are used, moderators should encourage consumers to explain these attributes to avoid misinterpretations (Grygorczyk et al. 2013). Furthermore, the moderator must emphasize the objective of this method (sensory description of the samples) and indicate the removal of hedonic‐related and imprecise terms during the discussion (Popoola et al. 2019).

Another limitation of the PAE method is that the significance of the differences is not determined, and the data analysis may be considered more complex than DA, which generally involves ANOVA, Tukey test, and PCA (da Costa et al. 2020). Most of the studies use the GPA method as the statistical approach. In this way, other methods could be investigated for confirmatory analysis. Finally, all participants should be present in the session, which may be difficult for the application of the test compared to other rapid methods that allow the evaluation individually by participants, such as CATA and JAR. However, time consumption for PAE is much lower than that of DA and other rapid sensory methods.

Although the number of studies using PAE has increased, more in‐depth information regarding its validity and reliability could make its application even more robust. This would help avoid overinterpretation of consumer‐generated data and ensure methodological rigor. Visalli et al. (2023) conducted an extensive study on the validity and reliability of dynamic (temporal) sensory methods, demonstrating the importance of understanding such parameters. In summary, what can be learned and applied to the present study involving PAE is that validity, in this context, refers to how accurately the method measures what it is intended to measure—namely, in the case of PAE, its ability to effectively capture the sensory attributes of a product that influence perception and acceptance. Reliability refers to the stability and consistency of the measurements since inconsistent results are likely to compromise the validity of the conclusions. Repeated testing enables repeatability assessment, that is, the consistency of results when the same test is applied to the same sample at different times. Although PAE has shown potential in discriminating between samples and generating consumer‐centered insights, further studies are needed to evaluate its internal consistency, sensitivity to product differences, and reproducibility across different contexts and consumer profiles.

## 6 Research Gaps

Methodological studies on the PAE method remain scarce and, therefore, represent an area to explore further, contributing significantly to understanding its robustness, applicability, and limitations across different contexts. For example, the performance of PAE could be compared with other descriptive methods involving consumers or even trained or semitrained assessors. Although PAE relies on consumer input, participants act as panelists and often receive some level of guidance or orientation. Thus, PAE could be compared with methods such as Classical DA, FL, PP, and Napping. These comparisons could consider aspects such as the richness and diversity of the attributes generated, the degree of discrimination between samples, and participant engagement.

Moreover, the role of the moderator during the discussion phase could be evaluated, comparing, for instance, the influence of a neutral moderator versus a more participative one and investigating the impact on the number, specificity, or type of attributes elicited.

Another relevant methodological aspect concerns session structure. It would be worthwhile to explore whether a single, longer session yields different data depth and quality results compared to shorter, distributed sessions—especially considering the potential effect of participant cognitive fatigue. It is also pertinent to examine how consumers’ prior sensory knowledge, such as previous experience with sensory evaluation methods or training in other descriptive approaches, affects the clarity, precision, and applicability of the attributes generated.

Another point worth exploring is using predefined vocabularies or attribute lists during the free description phase to support or stimulate participants’ thinking—a practice that may facilitate expression but also introduce bias. Furthermore, the number of samples evaluated per session is a factor that can influence both participant performance and the variety and consistency of elicited attributes. Investigating how many samples can be effectively assessed in a single session could help establish practical limits for applying the PAE method in different product contexts. Finally, studying the impact of scale length on PAE performance would be important.

The PAE method could also be expanded to target specific consumer segments, such as older adults, adolescents, children, and individuals with dietary restrictions (e.g., diabetics or those with food allergies or intolerances).

One major limitation of the PAE method is its predominant use in controlled laboratory environments. Due to the structured nature of the procedure—which involves moderated discussions, attribute elicitation, consensus‐building, and standardized product evaluations—its application in real‐world contexts, such as home‐use tests or natural consumption settings like cafés and restaurants, remains challenging. As a result, the insights generated may not fully reflect natural consumption behavior or contextual influences that shape sensory perception in everyday situations. The requirement for a trained moderator, standardized instructions, and group interaction limits the method's scalability and ecological validity. However, for home‐use contexts, virtual sessions may offer a promising alternative.

Another relevant limitation concerns the characterization of the participant sample. Although PAE involves consumers, the moderation and consensus processes may make them functionally closer to trained panelists. In addition, given the typically small sample sizes, the method does not allow for in‐depth evaluation of consumer‐intrinsic factors such as sociodemographic variables, attitudes, motivations, or consumption profiles. The lack of statistical power for subgroup analyses limits the understanding of how different consumer segments perceive product attributes. Future studies could explore ways to integrate the attribute elicitation phase into research with more extensive and diverse samples, expanding the interpretative reach of the results.

To date, most PAE applications have focused on fruits and derivatives, meat products, dairy products, dairy alternatives, and baked goods. Regarding emerging processing technologies, PAE has only been tested in the context of ohmic heating and ultrasound. Therefore, there is still great potential for applying the method to other food categories, such as savory snacks and extruded products, ready‐to‐eat meals, breakfast cereals, other plant‐based products, edible insect‐based items, hybrid products combining plant‐based and animal‐based ingredients, products with geographical indications, or foods developed from recycled ingredients and byproducts—as explored only in Da Silva (2021) using water‐soluble rice byproduct extract. Ethnic foods, such as the Coalho cheese evaluated in the study Soares et al. (2020), also represent a promising area. Moreover, there is room to use PAE to evaluate other emerging technologies, including microwave processing, radiofrequency, high‐pressure processing, supercritical fluids, and cold plasma.

## 7 Final Remarks

This review was the first to provide insights into applications of PAE in food products, as well as the challenges and trends. The PAE method has been applied mainly in dairy products and alternatives, suggesting opportunities for studies with fruit and derived products, meat products, and bakery goods. Generally, this method generated results similar to DA for the sensory characterization of food products using different processing techniques, ingredients, storage time, and fermentation length. Furthermore, it could compare the sensory profiles obtained for different consumer groups. A description of the method, consisting of six steps (acceptance test, attribute elicitation, attribute definition, scale generation, ranking of attributes, and sample evaluation), was presented and discussed, which is important, as standardization may help other researchers and food industries to apply the method. The moderator's ability, panelist number and type, and number of attributes and samples are important factors to consider. Opportunities for studies are highlighted, including comparing the acceptance of food products between PAE panelists and the general public, evaluating the PAE performance compared to DA in complex food matrices, and combining PAE with other sensory methods. This review brings important insights into the PAE method for researchers and food industries.

## Author Contributions

**Izabeli Batista Girarducci da Silva**: conceptualization, methodology, investigation, formal analysis, writing–original draft. **Marciane Magnani**: writing–original draft. **Erick Almeida Esmerino**: conceptualization, writing–original draft. **Elson Rogerio Tavares Filho**: conceptualization, writing–original draft, formal analysis, investigation, methodology. **Adriano Gomes Cruz**: conceptualization, investigation, formal analysis, writing–original draft. **Tatiana Colombo Pimentel**: conceptualization, methodology, investigation, validation, formal analysis, supervision, funding acquisition, visualization, project administration, resources, writing–original draft, writing–review and editing.

## Conflicts of Interest

The authors declare no conflicts of interest.
